# A Transcriptomic and Proteomic Analysis of the *Diaphorina citri* Salivary Glands Reveals Genes Responding to *Candidatus* Liberibacter asiaticus

**DOI:** 10.3389/fphys.2020.582505

**Published:** 2020-09-25

**Authors:** Xiao-Qiang Liu, Hong-Bo Jiang, Tian-Yuan Liu, Li Yang, Jia-Yao Fan, Ying Xiong, Tian-Xing Jing, Bing-Hai Lou, Wei Dou, Jin-Jun Wang

**Affiliations:** ^1^Key Laboratory of Entomology and Pest Control Engineering, College of Plant Protection, Southwest University, Chongqing, China; ^2^International Joint Laboratory of China-Belgium on Sustainable Crop Pest Control, Academy of Agricultural Sciences, Southwest University, Chongqing, China; ^3^Guangxi Key Laboratory of Citrus Biology, Guangxi Citrus Research Institute, Gulin, China

**Keywords:** *Diaphorina citri*, *Candidatus* Liberibacter asiaticus, salivary glands, comparative transcriptome, shotgun LC–MS/MS

## Abstract

The Asian citrus psyllid (ACP), *Diaphorina citri* Kuwayama, is the principal vector of the *Candidatus* Liberibacter asiaticus (CLas) bacterium that causes Huanglongbing (HLB) disease. The *D. citri* salivary glands (SG) is an important barrier to the transmission of CLas. Despite its importance, the transcriptome and proteome of SG defense against CLas are unstudied in *D. citri*. In the present study, we generated a comparative transcriptome dataset of the SG in infected and uninfected *D. citri* using an Illumina RNA-Seq technology. We obtained 407 differentially expressed genes (DEGs), including 159 upregulated DEGs and 248 downregulated DEGs. Functional categories showed that many DEGs were associated with the ribosome, the insecticide resistance, the immune response and the digestion in comparison with CLas-infected SG and CLas-free SG. Gene Ontology (GO) and Kyoto Encyclopedia of Genes and Genomes (KEGG) databases confirmed that metabolism and immunity were important functions in the SG. Among the DEGs, 68 genes (35 upregulated and 33 downregulated) encoding putative-secreted proteins were obtained with a signal peptide, suggesting that these genes may play important roles in CLas infection. A total of 673 SG proteins were identified in uninfected *D. citri* by liquid chromatography-mass spectrometry/mass spectrometry (LC-MS/MS) analysis, and 30 DEGs (15 upregulated and 15 downregulated) were found using the local tBLASTP programs. Among the 30 DEGs, many DEGs mainly involved in the metabolism and cellular processes pathways. This study provides basic transcriptome and proteome information for the SG in *D. citri*, and helps illuminate the molecular interactions between CLas and *D. citri*.

## Introduction

In phloem-feeding insects, saliva primarily originates from salivary glands (SG) and mediates the interaction between the insects and their host plants (Cherqui and Tjallingii, 2000). Saliva contains many bioactive components that function in food digestion, lubrication, tissue penetration and overcoming plant defenses (Hogenhout and Bos, 2011; Huang et al., 2016). Phloem-feeding Hemiptera have a specialized piercing-sucking stylet and a pair of SG, which secrete gelling and watery saliva during feeding (Sharma et al., 2014; van Bel and Will, 2016). The SG also act as a barrier to resist plant pathogen and virus transmission (Weintraub and Beanland, 2006; Hogenhout et al., 2008).

To characterize the functions of the SG or secreted saliva, the transcriptomes and/or proteomes of the SG and/or secreted saliva of phloem feeders were analyzed in several Hemiptera species such as *Acyrthosiphon pisum* (Carolan et al., 2011), *Nilaparvata lugens* (Ji et al., 2013), *Laodelphax striatellus*, *Sogatella furcifera* (Huang et al., 2018), *Bemisia tabaci* (Su et al., 2012), and *Diaphorina citri* (Yu and Killiny, 2018). The saliva of Hemiptera acts as a vector among insect–plant–pathogen interactions because insects produce saliva and inoculate pathogens into healthy plants during feeding (Weintraub and Beanland, 2006).

The Asian citrus psyllid (ACP), *Diaphorina citri* Kuwayama (Hemiptera: Liviidae), is a phloem-feeding pest of citrus. It is a principal vector of the *Candidatus* Liberibacter asiaticus (CLas) bacterium, which is the agent of Huanglongbing (HLB) (Grafton-Cardwell et al., 2013). HLB, also called citrus greening disease, is the most serious threat to the citrus industry worldwide, because prevention is difficult and there is no effective cure (Bové, 2006). Plant pathogens, in insect vectors, move from the alimentary canal into the hemolymph or other tissues (Hall et al., 2013). They then move into the SG, contaminate the salivary secretions and are injected into the host plant during feeding (Weintraub and Beanland, 2006; Hogenhout et al., 2008; Hall et al., 2013). The SG are the last, and most important, barrier in *D. citri* to resist the transmission of the HLB pathogen (Hogenhout et al., 2008). Among the tissues of CLas-infected *D. citri*, the proportion of infected (CLas-positive) SG was significantly lower than that of other tissues. This suggests that the SG may act as a barrier to reduce CLas transmission (Ammar et al., 2011). The SG-secreted proteins from other insect phloem feeders act as effector proteins that facilitate their feeding or repress host immune responses, like C002 (Mutti et al., 2008), NlEG1 (Ji et al., 2017), Bt56 (Xu et al., 2019), and macrophage migration inhibitory factor (MIF) (Naessens et al., 2015). Although the saliva of *D. citri* has been analyzed using LC-MS/MS (Yu and Killiny, 2018), the differences of SG components in CLas-infected and uninfected *D. citri* and the functions of these different components are unknown. SG are important in *D. citri* for feeding, responding to host plant defenses and barring pathogens. This motivated our study of the different gene and protein repertoires of SG in CLas-infected and uninfected *D. citri*.

**FIGURE 1:**
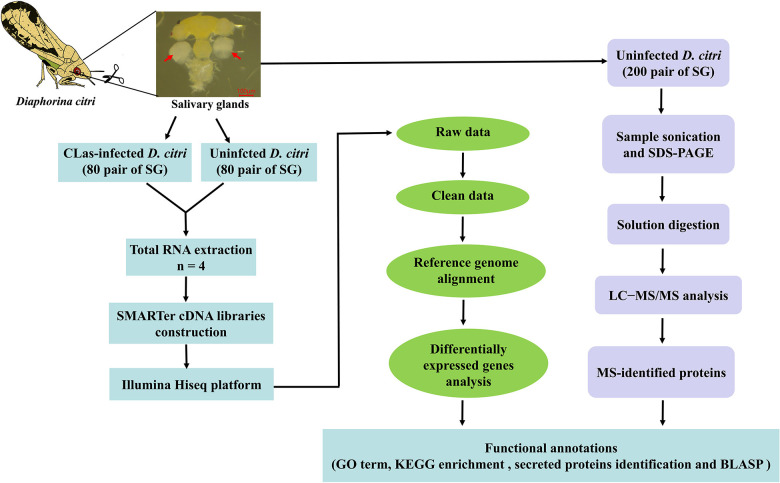
Flowchart showing the methodology used for this study.

We reported the first transcriptomic sequences from the SG of CLas-infected *D. citri* and uninfected *D. citri*. Comparative transcriptomes were generated to analyze differential expressed genes (DEGs) using RNA sequencing (RNA-seq). Our goal was to find genes in *D. citri* SG responding to CLas. To identify proteins potentially acting as salivary effectors, detoxification enzymes and digestive enzymes, we also conducted proteomic analyses of the SG of uninfected *D. citri* using LC-MS/MS. This study provides valuable transcriptomic and proteomic data for further studies of functional gene activity in the SG and understanding of the molecular interactions between CLas and *D. citri*. Overall experimental workflow used in this study was shown in Figure 1.

## Materials and Methods

### ACP Rearing and SG Collection

The CLas-infected *D. citri* strain was originally collected in an abandoned citrus orchard at Tandong town, Zhanggong district, Ganzhou city, Jiangxi Province, China. The infected psyllid population and laboratory *D. citri* strain were, respectively, maintained on CLas-infected or uninfected Newhall naval orange *Citrus sinensis* Osbeck in insect rearing cages (60 cm × 60 cm × 90 cm) at Gannan Normal University, Ganzhou city, Jiangxi Province, China. The *D. citri* were reared at 27 ± 1°C with 70 ± 5% relative humility under a 14:10 h (L:D) photoperiod. To confirm the rate of CLas-infected *D. citri* population, genomic DNA from the thorax of a single *D. citri* was extracted, and specific primer set OI1/OI2c was used to detect the exist of HLB pathogen. The result of agarose gel showed that the proportion of HLB carried by CLas-infected *D. citri* population was up to 80% (Supplementary Figure S1). The male and female adults of CLas-infected and uninfected strains were anesthetized on ice, and their SG were dissected out in cold 1 × phosphate-buffered saline (PBS). Each sample contained approximately 80 pairs of SG, and four biological replicates of the two psyllid strains were prepared.

### Total RNA Extraction and cDNA Library Construction

Total RNA was extracted from eight samples using Trizol regent (Invitrogen, Carlsbad, CA, United States) according to manufacturer protocol. The integrity of the RNA was monitored on 1% agarose gels, and the quality was assessed using the RNA Nano 6000 Assay Kit of the Bioanalyzer 2100 system (Agilent Technologies, CA, United States). The SMARTer cDNA libraries were constructed from SG using the SMARTer Ultra^TM^ Low Input RNA Kit (Clontech, Mountain View, CA, United States) following manufacturer’s instructions (Picelli et al., 2013).

### Illumina Sequencing

Sequencing libraries were generated using an NEBNext^®^ Ultra^TM^ Directional RNA Library Prep Kit for IlluminaR (NEB, United States) following manufacturer’s recommendations. To select cDNA fragments 150–200 bp in length, the library fragments were purified with the AMPure XP system (Beckman Coulter, Beverly, MA, United States). PCR products were purified (AMPure XP system), and library quality was assessed on an Agilent Bioanalyzer 2100 system. After cluster generation, the library preparations were sequenced on an Illumina Hiseq platform (Novogene Bioinformatics Technology Co., Ltd., Tianjin, China), and 125/150 bp paired-end reads were generated.

### Data Filtering and Reads Mapping

Clean reads were obtained by removing reads containing adapters, ploy-N and low-quality reads from the raw data. Reference genome and gene model annotation files were downloaded directly from the genome website^1^. We used Hisat2 v2.0.5^2^ to build the index of the reference genome and align the paired-end clean reads with the reference genome (Trapnell et al., 2009). Then, HTSeq v0.6.0 was used to count the read numbers mapped to each gene. Fragments Per Kilobase per Million (FPKM) of each gene was calculated based on the length of the gene and reads count mapped to this gene.

### Analysis of Differentially Expressed Genes

EdgeR R package (3.12.1) was used to perform differential expression analysis of the two strains. The resulting *P*-values were adjusted using the Benjamini and Hochberg approach for controlling the false discovery rate. Genes with an adjusted *P*-value < 0.05 and an absolute value of log_2_ (Fold change) ≥ 1 found by DESeq.2 were assigned as differentially expressed (Love et al., 2014). Gene Ontology (GO) enrichment analysis of differentially expressed genes (DEGs) was implemented by the GOseq, in which the gene length bias was corrected. GO terms with a corrected *P*-value less than 0.05 were considered to be significantly enriched by DEGs. All of the DEGs were assigned to the Kyoto Encyclopedia of Genes and Genomes (KEGG) database. KOBAS software was used to test the statistical enrichment of differential expression genes in KEGG pathways^3^. To identify potential SG-secreted proteins of DEGs, the signal peptide was determined using the SignalP 4.1 Server^4^.

### RT-qPCR Validation

To confirm the reliability of transcriptome data, 10 DEGs (five upregulated and five downregulated) were randomly selected to analyze the transcript levels using RT-qPCR. Because it was difficult to dissect the SG and obtain large amounts of RNA, about 100 ng total RNA of each sample was used to synthesize the first-strand cDNA using the PrimeScript RT reagent Kit with gDNA Eraser (Takara, Dalian, China). Primer 3.0 software^5^ was used to design specific RT-qPCR primers. The amplification efficiency of each primer was 90–110%, and all of the primers used are listed in Supplementary Table S1. NovoStar SYBR qPCR SuperMix (Novoprotein Scientific, Shanghai, China) was used in RT-qPCR and the CFX384 Real-time System (Bio-Rad, Singapore) was conducted as previously described (Liu et al., 2019). The reference genes *DcitActin1* (XM_008473151) and *DcitGAPDH2* (XM_008481620) were used to standardize the relative quantities using the 2^–ΔΔ^*^*C*^*^*T*^ method (Livak and Schmittgen, 2001). The data were analyzed by an independent sample *t*-test using SPSS 16.0 software (SPSS Inc., Chicago, IL, United States). Statistically significant differences were considered to be *P* < 0.05 or *P* < 0.01.

### Protein Preparation and Digestion

For the protein assay, 200 pairs of SG from the uninfected *D. citri* strain were dissected out in SDT lysis buffer (4% SDS, 100 mM DTT, 150 mM Tris–HCl, pH 8.0). SG were homogenized in SDT buffer and sonicated at 4°C by 5 × 10-s bursts, and then boiled for 10 min. The homogenate was centrifuged at 14,000 *g*, 4°C for 30 min, and the quality of the supernatant solution was checked by 12.5% sodium dodecyl sulfate-polyacrylamide gel electrophoresis (SDS–PAGE). Protein digestion was performed based on the manufacturer’s protocol. Briefly, 100 μg of proteins were concentrated with a 3-kDa microcon unit and alkylated with 100 μL of iodoacetamide buffer (100 mM IAA in UA) for 30 min in the darkness. The detergent, DTT and other low-molecular-weight components were removed using 100 μL UA buffer (8 M Urea, 150 mM Tris–HCl pH 8.0) by repeated ultrafiltration twice, and the sample was washed with 100 μL 25 mM NH_4_HCO_3_ buffer twice. The protein suspensions were digested with 2 μg trypsin (Promega) in 40 μL 25 mM NH_4_HCO_3_ buffer for 16–18 h at 37°C, and the resulting peptides were collected as a filtrate. After centrifugation, the peptides were desalted on Empore^TM^ SPE Cartridges C18 (Thermo Scientific, Rockford, IL, United States) concentrated by vacuum drying and reconstituted in 40 μL of 0.1% (v/v) formic acid. The peptide content was estimated by UV light spectral density at 280 nm.

### LC-MS/MS Analysis

LC-MS/MS analysis was conducted on a Q Exactive mass spectrometer (Thermo Fisher Scientific, MA, United States) coupled with Easy nLC (Thermo Fisher Scientific) (Shanghai Applied Protein Technology Co., Ltd., Shanghai, China). The mass spectrometer was operated in a positive ion mode. The chromatographic system contained a trap column (2 cm × 100 μm, 5 μm-C18) and an analytical column (100 mm × 75 μm, 3 μm-C18). After trap column equilibration with 97% buffer A (0.1% formic acid), 5 μL digested peptides were eluted by a linear gradient of buffer B (0.1% formic acid and 84% acetonitrile) for 120 min at a flow rate of 300 nL/min. Further MS/MS analysis of fractions was carried out on Q Exactive mass spectrometer for 120 min. MS data were acquired to select the 20 most abundant ions from the full scan (300–1,800 m/z) for HCD fragmentation at a resolution of 17,500 at m/z 200, and an isolation width of 2 m/z, and 30% collision energy. The dynamic exclusion duration was 60 s. Survey scans for MS1 were acquired at a resolution of 70,000 at m/z 200. For protein identification, the following options were used: 20 ppm for peptide mass tolerance, 0.1 Da for MS/MS tolerance, two missed cleavages allowed, carbamidomethyl (C) as a fixed modification and oxidation (M) and acetyl (protein N-term) as variable modifications. Additionally, an automatic decoy database search was conducted with false discovery rate (FDR) ≤ 0.01. MS/MS spectra were searched against the *D. citri* genome, including 29,287 proteins^6^ using the MASCOT engine (Matrix Science, London, United Kingdom, version 2.2), assuming digestion with trypsin.

### Presence of the DEG in Proteomic Data

Additionally, a correlated analysis of all of the DEGs related to shotgun data was performed using the local tBLASTp program, with an *E*-value < 1.0E^–50^ and a bit score >100. All of the identifications were manually validated and selected based on the score, *E*-value and identities (%).

## Results

### Sequencing Quality

To evaluate sequencing quality, we used FastQC software to assess the quality of each sample before and after adaptor/sequences trimming. Totals of 50.1–56.0 million and 53.0–65.6 million raw reads were generated using an Illumina HiSeq from the SG of uninfected and CLas-infected *D. citri*, respectively (Table 1). After removing the low-quality reads, 40.7–46.4 and 42.4–53.0 million reads were retained for the following assembly. We also tested all of the samples using FastQC for GC content, Q20 (Phred score > 20) and Q30 (Phred score > 30). Overall, mean values of 51.88% and 80.18% filtered RNA-seq reads were subsequently mapped to the *D. citri* genome data (Table 2). These results suggested that the sequencing quality was adequate.

**TABLE 1:** Quality control and data statistics of two salivary gland transcriptomes in *Diaphorina citri.*

**Sample**	**SG_CK1**	**SG_CK2**	**SG_CK3**	**SG_CK4**	**SG_HLB1**	**SG_HLB2**	**SG_HLB3**	**SG_HLB4**
Total reads	50,103,538	53,220,848	54,0724,94	56,020,608	65,581,366	63,015,308	53,032,528	57,692,402
Total clean reads	40,699,876	44,384,444	43,522,284	46,353,188	52,959,882	49,525,062	42,370,648	45,166,030
GC percent (%)	39.18	38.92	40.05	38.88	43.56	43.19	42.94	42.92
Q20 (%)	97.82	97.83	97.85	97.87	97.70	97.80	97.87	97.60
Q30 (%)	93.32	93.30	93.44	93.42	93.16	93.43	93.58	92.88

**TABLE 2:** Summary reads mapping of two salivary gland transcriptomes in *D. citri.*

**Sample**	**SG_CK1**	**SG_CK2**	**SG_CK3**	**SG_CK4**	**SG_HLB1**	**SG_HLB2**	**SG_HLB3**	**SG_HLB4**
Total map	21,630,889	21,863,502	25,475,704	21,600,876	42,839,314	39,384,904	34,006,639	36,148,814
(%)	(53.15)	(49.26)	(58.53)	(46.6)	(80.89)	(79.53)	(80.26)	(80.04)
Multiple map	5,621,779	5,573,538	6,739,118	6,280,554	11,008,895	10,405,531	8,741,489	9,127,659
(%)	(13.81)	(12.56)	(15.48)	(13.55)	(20.79)	(21.01)	(20.63)	(20.21)
Unique map	16,009,110	16,289,964	18,736,586	15,320,322	31,830,419	28,979,373	25,265,150	27,021,155
(%)	(39.33)	(36.70)	(43.05)	(33.05)	(60.10)	(58.51)	(59.63)	(59.85)
Read map 1	8,072,390	8,198,349	9,441,878	7,717,414	16,033,979	14,592,735	12,723,938	13,611,109
(%)	(19.83)	(18.47)	(21.69)	(16.55)	(30.28)	(29.47)	(30.03)	(30.14)
Read map 2	7,936,720	8,091,615	9,294,708	7,602,908	15,796,440	14,386,638	12,541,212	13,410,046
(%)	(19.50)	(18.23)	(21.36)	(16.40)	(29.83)	(29.05)	(29.60)	(29.69)

### Function Annotation of Differentially Expressed Genes

**FIGURE 2:**
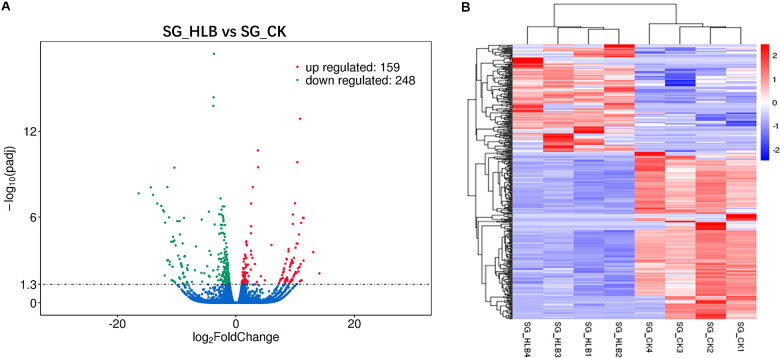
Volcano plot **(A)** and hierarchical cluster analysis **(B)** of differential expressed genes (DEGs). **(A)** The ordinate shows the significance level of difference in DEGs, and the abscissa represents the fold change of gene expression in different samples (log_2_ Fold change), respectively. Red, green, and blue points indicate upregulated, downregulated, and no significant difference genes, respectively. **(B)** Rows represent different DEGs, and columns represent different samples between CLas-infected and uninfected *Diaphorina citri*. Red color and purple color suggest high and low gene expression level, respectively.

A comparative gene expression analysis between CLas-infected and CLas-uninfected *D. citri* was performed by DESeq.2, with an adjusted *P*-value < 0.05 and an absolute value of log_2_ (Fold change) ≥ 1. Differential expression analysis showed significant differences in CLas-infected *D. citri* compared with uninfected *D. citri*. As a result, 20,690 genes were obtained in the transcriptomes of *D. citri* SG. A total of 407 DEGs showed significant changes; 159 genes were upregulated and 248 genes were downregulated compared with that in the control (Figure 2A). In addition, the hierarchical clustering of all of the DEGs was conducted to determine the expression patterns of the identified genes (Figure 2B). Moreover, many DGEs were classified and associated with the ribosome, the insecticide resistance, the immune response and the digestion (Supplementary Table S2). Among these DEGs, ribosomal protein (two upregulated and four downregulated), glutathione *S*-transferases (GSTs, two upregulated and one downregulated), UDP-glucuronosyltransferases (UGTs, two upregulated and one downregulated), cytochrome P450s (CYPs, five downregulated), cathepsins (one upregulated and seven downregulated) showed significant variation (Supplementary Tables S2 S3).

**FIGURE 3:**
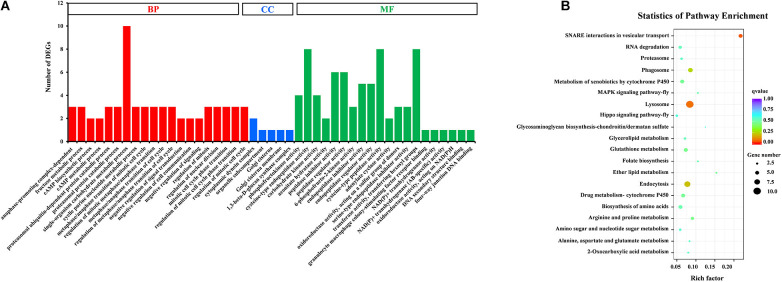
GO classification **(A)** and KEGG enrichment analysis **(B)** of DEGs. **(A)** The results were functionally grouped into three categories: BP (biological process), CC (cellular component), and MF (molecular function). For each categories, the top 20 GO terms were selected for functional analysis. The *y*-axis indicates the number of DEGs. **(B)** The top 20 of pathways were functionally analyzed in the KEGG. The *X*-axis indicates the gene ratio. The *Y*-axis indicates different pathways.

Gene Ontology (GO) assignments were used to classify the functions of DEGs, and a *P*-value < 0.05 was deemed to indicate significant enrichment. Of these DEGs, 105 genes (25.8%) significantly corresponded to at least one GO term (Supplementary Table S4). The GO terms were functionally grouped into three categories: biological processes, cellular component, and molecular function. For each category, the 20 most significantly enriched GO terms (Top 20) were selected for further analysis (Figure 3A). For biological processes, single-organism carbohydrate metabolic process was the most abundant with 10 categories. The cellular component only included five groups with six categories. For the cellular component, cysteine-type endopeptidase activity, cysteine-type peptidase activity and transferase activity-transferring hexosyl groups were the most abundant of 24 categories. To identify the biological pathways that were active in the SG of *D. citri*, all DEGs were assigned to the reference canonical pathways in KEGG. A total of 177 DEGs were matched separately with 57 pathways (Supplementary Table S5), and the top 20 of these were shown in Figure 3B. Among these pathways, SNARE interactions in vesicular transport (5 transcripts), lysosome (10 transcripts), endocytosis (eight transcripts), and phagosome (six transcripts) were the most enriched pathways. Based on GO and KEGG annotations, the SG are active in metabolism, which confirmed that metabolism is important in the SG.

In addition, to identify potential SG-secreted proteins, the N-terminal signal peptides were predicted using the SignalP 4.1 Server. In total, 68 SG-secreted proteins were obtained from DEGs (Supplementary Table S6). Among these, 35 secreted proteins were significantly upregulated and 33 were significantly downregulated in CLas-infected *D. citri* compared with the uninfected control. More interestingly, the transcript of multiple secreted proteins related to digestion exhibited significant downregulation, including cathepsins, maltases, salivary cysteine-rich peptide and aminopeptidase (Supplementary Table S6).

### RT-qPCR Validation

**FIGURE 4:**
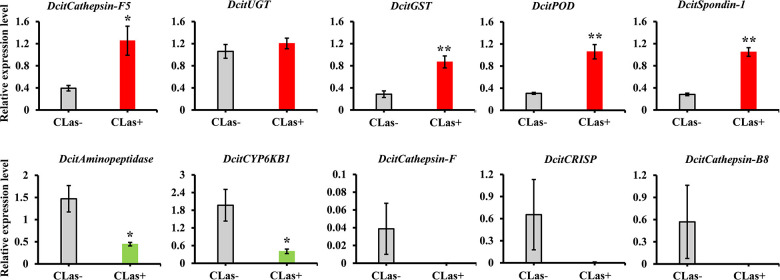
The expression analysis of 10 DEGs to validate the RNA-Seq data by RT-qPCR. Relative expression levels were normalized to the reference genes *GAPDH2* and *Actin1* expression levels using the 2^– ΔΔ^*^*C*^*^*t*^ method. The vertical bars represent standard errors (SE) of the mean (*n* = 4) and asterisks represent the significant difference (*P < 0.05 or **P < 0.01) using independent *t-*test.

To validate the transcriptome data, we checked the transcript abundance of 10 DEGs (five upregulated and five downregulated) using RT-qPCR. The information of selective genes is listed in Supplementary Table S7. As expected, the results of RT-qPCR were basically consistent with our RNA-Seq data (Figure 4). For example, the genes of glutathione *S*-transferase, peroxidase and spondin-1 showed significant increases in CLas-infected SG in both RT-qPCR and transcriptome analysis, with a consistent level of upregulation. In addition, five genes were downregulated in RT-qPCR analysis, but only two genes (Aminopeptidase and CYP6KB1) had significant downregulation. Overall, the RT-qPCR results confirmed the reliability of the transcriptome data.

### Proteins Identified in the SG of *D. citri*

For protein validation, high peptide confidence was extracted, and an automatic decoy database search was conducted with FDR ≤ 0.01. In total, 673 SG proteins of *D. citri* were identified by shotgun LC-MS/MS analysis. The protein with 1, 2 and ≥3 unique peptides were deemed as low, medium and high expression, respectively. Thus, significant identification was contained when at least two unique peptides. Among these proteins, 369 proteins had high or medium enrichment with 39-2 unique peptides, while 304 proteins exhibited low abundance with one unique peptide (Supplementary Table S8). Sequence coverage and molecular weight is listed in Supplementary Table S8. Additionally, 84 salivary proteins with signal peptide were identified using SignalP 4 Server (Supplementary Table S9).

### Presence of the DEGs in Proteomic Data

**FIGURE 5:**
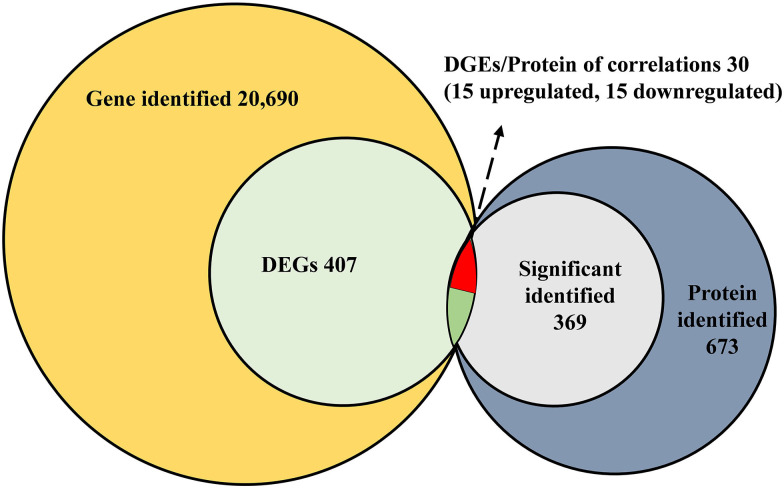
Correlated analysis between DEGs of RNA-seq and protein of shotgun. The local tBLASTp program was performed to analyze the correlation with an *E*-value less than 1.0E^– 50^ and a bit score >100.

All of the DEGs were compared against the protein sequences for homology searching through a local tBLASTP search. A total of 30 DEGs were found in the proteome, and the BLAST result is shown in Table 3. The correlation between DEGs and shotgun data is shown in Figure 5. Among these DEGs, 15 genes were significantly upregulated and 15 genes were significantly downregulated. Additionally, these genes were annotated to the KEGG pathway (Table 3), and the result showed that many DEGs involved in the metabolism and cellular processes pathways.

**TABLE 3:** Presence of the DEGs in proteomic data by a local tBLASTP search.

**Gene ID (DEGs)**	**KEGG pathway**	**Log_2_ fold**	**Query length (aa)**	**Score**	***E*-value**	**Identifies (%)**	**tBLASTP annotation (*Diaphorina citri*)**
DcitrP012115.1.1	–	–1.18	463	946	0.0	100	XP_008476451.1 protein odr-4 homolog
DcitrP030815.1.1	Arginine and proline metabolism	1.53	738	838	0.0	78	XP_026681313.1 delta-1-pyrroline-5-carboxylate synthase-like
DcitrP033650.1.1	–	1.31	896	1298	0.0	86	XP_026686168.1 mucin-5AC-like isoform X2
DcitrP037890.1.1	–	1.16	290	577	0.0	98	XP_026684327.1 uncharacterized protein LOC103516011
DcitrP054635.1.1	Endocytosis	–1.21	586	738	0.0	66	XP_008469508.1 EH domain-containing protein 1
DcitrP067050.1.1	Cysteine and methionine metabolism	–2.44	517	1078	0.0	99	XP_026680915.1 DNA (cytosine-5)-methyltransferase PliMCI-like
DcitrP081725.1.1	–	1.02	298	564	0.0	99	XP_017304024.1 coiled-coil domain-containing protein 1-like
DcitrP091755.1.1	Ribosome	1.45	578	627	0.0	99	Q0PXX8.1 40S ribosomal protein SA
DcitrP094660.1.1	–	1.0	438	707	0.0	91	XP_017304347.1 uncharacterized protein LOC103521618
DcitrP030830.1.1	Arginine and proline metabolism	1.37	774	391	7e-134	100	XP_008483053.2 probable delta-1-pyrroline-5-carboxylate synthase
DcitrP005130.1.1	Hippo signaling pathway– fly	–1.15	125	188	1e-62	74	XP_017301022.1 14-3-3 protein epsilon
DcitrP008395.1.1	Hippo signaling pathway– fly	–1.27	602	203	5e-65	100	XP_008479623.1 14-3-3 protein zeta-like
DcitrP015560.1.1	MAPK signaling pathway– fly	–1.55	126	184	2e-63	99	XP_026678415.1 profilin, partial
DcitrP020605.1.1	Glutathione metabolism	1.55	217	382	3e-139	100	glutathione *S*-transferase-like protein, partial
DcitrP022120.1.1	–	2.84	383	104	8e-71	100	XP_008483284.2 uncharacterized protein LOC103519971
DcitrP023525.1.1	Protein processing in endoplasmic reticulum	–4.55	186	327	5e-118	85	XP_008475973.1 alpha-crystallin A chain-like
DcitrP024125.1.1	–	–1.86	136	248	1e-86	97	XP_017303686.1 histone H3-like, partial
DcitrP040835.1.1	–	–1.21	233	311	6e-112	99	Vesicle-associated membrane protein-associated protein A
DcitrP041665.1.1	Peroxisome	1.65	310	474	2e-170	72	XP_008485007.1 isocitrate dehydrogenase [NADP] cytoplasmic
DcitrP042945.1.1	Protein processing in endoplasmic reticulum	1.46	294	416	8e-149	100	Dolichyl-diphosphooligosaccharide-protein glycosyltransferase 48 kDa subunit-like
DcitrP044485.1.1	–	–1.44	120	201	3e-70	96	XP_008484707.1 histone H2B-like
DcitrP045865.1.1	FoxO signaling pathway	–2.28	166	332	4e-120	98	XP_008468841.1 GTPase HRas
DcitrP051350.1.1	Ether lipid metabolism	–1.92	283	424	5e-153	81	XP_026678696.1 putative phosphatidate phosphatase
DcitrP051375.1.1	–	1.05	289	224	5e-76	97	XP_017299158.1 glyoxalase domain-containing protein 4-like
DcitrP059375.1.1	Endocytosis	–1.28	182	375	3e-137	100	XP_026676609.1 ADP-ribosylation factor 1
DcitrP068670.1.1	–	2.58	140	256	3e-91	95	XP_008485458.1 uncharacterized protein LOC103522132
DcitrP078265.1.1	SNARE interactions in vesicular transport	–1.16	213	434	2e-159	100	Vesicle-trafficking protein SEC22b-B
DcitrP081735.1.1	–	1.06	279	341	1e-121	99	XP_008469045.1 uncharacterized protein LOC103506434
DcitrP081740.1.1	–	1.33	223	452	3e-166	99	XP_017298359.2 uncharacterized protein LOC103506419 isoform X2
DcitrP088680.1.1	Mitophagy – animal	–1.90	208	159	1e-51	100	XP_026682559.1 ras-related protein Rab-7a

## Discussion

The salivary glands as a crucial secretary tissue is important in insect feeding and defense against pathogens. We found differences in the transcriptomic levels of SG from CLas-infected and uninfected *D. citri*. The comparative transcriptome analysis showed that 68 differentially expressed genes in the SG of CLas-infected and uninfected *D. citri* could be involved in metabolism and immunity processes. However, the number of secreted proteins might be underestimated due to missing signal peptide sequences from partial sequences. In *N. lugens*, 67 unigenes encoding putative secretory proteins were obtained in a comparison of the SG DEGs of two *N. lugens* populations with different virulence (Ji et al., 2013). These results are comparable with the numbers of putative secreted proteins of our results. The SG continue to produce proteins during insect feeding, and thus, have a high level of metabolic and immune activity. An enrichment of genes in the SG related to protein synthesis, transport and energy metabolism has also been observed in other Hemiptera, such as *B. tabaci* (Su et al., 2012), *N. lugens* (Ji et al., 2013), *S. furcifera* and *L. striatellus* (Huang et al., 2018), and *Empoasca fabae* (DeLay et al., 2012). These results suggest that the SG have conserved biological functions in different insect species.

Transmission of CLas depends on circulation and replication of the bacterium in the hemolymph and overcoming the SG barrier in *D. citri*. The mechanisms that facilitate immune defenses of *D. citri* to CLas in the SG are unknown. In this study, the comparative transcriptome data suggest that many DEGs are involved in immune defenses, including the lysosome pathway, endocytosis pathway and phagosome pathway. Among these pathways, multiple cathepsins were significantly altered in CLas-infected *D. citri*, suggesting that these proteins might be important in defense against CLas. The cathepsins are involved in processes like development, growth, metamorphosis, apoptosis and immunity (Saikhedkar et al., 2015). For example, two *D. citri* cathepsin genes were significantly induced by CLas, suggesting that these genes play an important role in the immune response (Yu et al., 2019). In addition, cathepsin is a cysteine protease and functions as a primary digestive enzyme to degrade host defense proteins. It is common in the watery saliva of *Diuraphis noxia* (Nicholson et al., 2012), *N. lugens* (Liu et al., 2016) and *D. citri* (Yu and Killiny, 2018). Our results showed that six cathepsins with signal peptides were significantly changed (one upregulated and five downregulated) in CLas-infected *D. citri*. This result indicated that cathepsins may act as saliva proteins involved in the host defense response. The digestive enzymes like the majority of cathepsins, salivary cysteine-rich peptides, aminopeptidases and maltases were significantly downregulated in CLas-infected *D. citri*, suggesting that the digestive ability of CLas-infected *D. citri* was reduced due to the CLas infection. For example, knocking down a digestive enzyme gene (*NlEG1*) caused a decrease in the ability to reach the phloem and ingest food in *N. lugens* (Ji et al., 2017). Additionally, previous study reported that CLas-infected *D. citri* had significantly decreased the non-probing time, salivation time and phloem ingestion time compared with uninfected *D. citri*. This indicated that CLas-infected *D. citri* tends to forage more often (Killiny et al., 2017). Our data provide some evidence, at the molecular level, why CLas-infected *D. citri* have reduced feeding capacity.

Insect detoxification enzymes, such as cytochrome P450s (CYPs) and glutathione *S*-transferases (GSTs), participate in the metabolism of xenobiotics (Li et al., 2007). *CYPs* and *GSTs* are ubiquitous in the SG of different insects. For example, 59 *CYPs* and 20 *GSTs* genes are present in the SG of *Nephotettix cincticeps*, and these have various expression levels (Matsumoto et al., 2014). While eight *CYP* and five *GST* genes were found in *B. tabaci* (Su et al., 2012), only one and three were found in *A. pisum* (Carolan et al., 2011). However, little is known on the role of *CYP* and *GST* genes in the insect SG. In two *N. lugens* populations with different virulence, 12 *CYP* genes were involved in the drug metabolism pathway. Four were upregulated and eight were downregulated in the SG of the Mudgo population relative to those of the TN1 population (Ji et al., 2013). These data indicate that DEGs play important roles in metabolic response of SG against viral infection. In our DEGs, only two *GSTs* genes were significantly upregulated, but one *GST* and five *CYP* genes were significantly downregulated in CLas-infected *D. citri*. Similar results were found in another *D. citri* study where the enzyme activities of CYPs and GSTs were significantly lower in CLas-infected adults than in uninfected *D. citri* adults (Tiwari et al., 2011). CLas infection may suppress the transcriptional expression of *CYPs* and *GSTs*, resulting in reduced activities of these detoxification enzymes. Other detoxification enzymes, such as UDP-glucuronosyltransferases (UGTs), were also reported to contribute to the metabolism of xenobiotics in *D. citri* (Tian et al., 2019). Two UGT genes with signal peptides were found in the DEGs, and they were significantly upregulated in the SG of CLas-infected *D. citri*. UGTs may be involved in defending against CLas infection in the SG.

Our shotgun LC-MS/MS data identified 673 SG proteins in CLas-free *D. citri*. Significant identification of 369 proteins was obtained with at least two unique peptides. In *Schlechtendalia chinensis*, 141 proteins were obtained from SG by an LC-MS/MS analysis (Yang et al., 2018). A similar result was found in the SG proteomes of *Nezara viridula* and *Halyomorpha halys*, identifying 305 and 238 proteins, respectively (Serteyn and Francis, 2019). Compared with these studies, we identified slightly more proteins in the SG of *D. citri*, and our data provide valuable information for further study of functional protein activity in the SG. In addition, 86 saliva proteins were identified from *D. citri* via an LC-MS/MS analysis (Yu and Killiny, 2018). More proteins were obtained from the SG than from the gathered saliva in *D. citri*. However, we did not perform a comparative analysis of the SG proteomes of *D. citri* and the SG/saliva proteomes of other Hemiptera species. The majority of protein species identified in Hemiptera are analogous. Our results will be useful for identifying potential functional proteins like salivary effectors and provide a framework for future studies on the molecular interactions between phloem-feeding insects and host plants.

## Conclusion

We conducted a comparative, transcriptomic-level analysis of the SG of CLas infected and uninfected *D. citri*. The SG were metabolically active and hundreds of SG proteins in CLas uninfected *D. citri* were identified at the proteomic level. Our study is the first to analyze the SG of *D. citri* basing on the combination of comparative transcriptomics and proteomics, which can be further utilized for the identification of functional SG proteins. In addition, this work provides valuable transcriptomic and proteomic information about the molecular interactions between CLas and *D. citri*.

## Data Availability Statement

The raw read files from eight samples were uploaded to the NCBI Sequence Read Archive (SRA) database under the accession numbers SRR11801814–SRR11801821 (http://www.ncbi.nlm.nih.gov/sra). The mass spectrometry proteomics data were deposited in the ProteomeXchange database (http://proteomecentral.proteomexchange.org) via the iProX partner repository (Ma et al., 2019) with the dataset identifier PXD019624.

## Author Contributions

H-BJ, WD, and J-JW conceived and designed the project. T-YL and YX dissected salivary gland samples. X-QL, J-YF, and T-XJ contributed to the analysis of data. X-QL and LY wrote the manuscript. X-QL, H-BJ, and J-JW revised the manuscript. All authors contributed to the article and approved the submitted version.

## Conflict of Interest

The authors declare that the research was conducted in the absence of any commercial or financial relationships that could be construed as a potential conflict of interest.

## Footnotes

1 ftp://ftp.citrusgreening.org/genomes/Diaphorina_citri/assembly/

2 https://anaconda.org/biobuilds/hisat2

3 http://www.genome.jp/kegg/

4 http://www.cbs.dtu.dk/services/SignalP/

5 http://primer3.ut.ee

6 http://www.uniprot.org

## Supplementary Material

FIGURE S1 RT-PCR detection the proportion of *Candidatus* Liberibacter asiaticus (CLas) in CLas-infected *Diaphorina citri* population. The thorax from a single *D. citri* was dissected, then genomic DNA was isolated, and RT-PCR was performed using the OI1/OI2c primer (forward primer 5′-GCGCGTATGCAATACGAGCGGCA-3′ and reverse primer 5′-GCCTCGCGACTTCGCAACCCAT-3′). Twenty *D. citri* were randomly selected to check HLB, and 16 ones exhibited a clear OI1 band with 1160 bp in length.

TABLE S1 Primers used in RT-qPCR for validation of DEGs.

TABLE S2 Differentially expressed genes upon to CLas infection involved in the ribosome, the insecticide resistance, the immune response and the digestion in comparison with CLas-infected SG and CLas-free SG.

TABLE S3 Summary of all differentially expressed genes.

TABLE S4 Gene Ontology (GO) annotation of DEGs from the salivary gland transcriptomes between CLas infected and CLas uninfected *Diaphorina citri*.

TABLE S5 Kyoto Encyclopedia of Genes and Genomes (KEGG) enrichment analysis of DEGs from the salivary gland transcriptomes between CLas infected and CLas uninfected *D. citri*.

TABLE S6 Differentially expressed genes (DEGs) with a putative signal peptide.

TABLE S7 The information of selected 10 DEGs used in RT-qPCR validation.

TABLE S8 The salivary gland proteins of CLas free *D. citri* identified by shotgun LC-MS/MS analysis.

TABLE S9 The putative secretory salivary gland proteins from proteome.

## References

[B1] Ammar E. D. Shatters R. G. Lynch C. Hall D. G. (2011). Detection and relative titer of *Candidatus* Liberibacter asiaticus in the salivary glands and alimentary canal of *Diaphorina citri* (Hemiptera: Psyllidae) vector of citrus Huanglongbing disease. *Ann. Entomol. Soc. Am.* 104 526–533. 10.1603/an10134

[B2] Bové J. M. (2006). Huanglongbing: A destructive, newly-emerging, century-old disease of citrus. *J. Plant Pathol.* 88 7–37. 10.4454/jpp.v88i1.828 32896216

[B3] Carolan J. C. Caragea D. Reardon K. T. Mutti N. S. Dittmer N. Pappan K. (2011). Predicted effector molecules in the salivary secretome of the pea aphid (*Acyrthosiphon pisum*): a dual transcriptomic/proteomic approach. *J. Proteome Res.* 10 1505–1518. 10.1021/pr100881q 21226539

[B4] Cherqui A. Tjallingii W. F. (2000). Salivary proteins of aphids, a pilot study on identification, separation and immunolocalisation. *J. Insect Physiol.* 46 1177–1186. 10.1016/S0022-1910(00)00037-8 10818245

[B5] DeLay B. Mamidala P. Wijeratne A. Wijeratne S. Mittapalli O. Wang J. (2012). Transcriptome analysis of the salivary glands of potato leafhopper. *Empoasca fabae. J. Insect Physiol.* 58 1626–1634. 10.1016/j.jinsphys.2012.10.002 23063500

[B6] Grafton-Cardwell E. E. Stelinski L. L. Stansly P. A. (2013). Biology and management of Asian citrus psyllid, vector of the huanglongbing pathogens. *Annu. Rev. Entomol.* 58 413–432. 10.1146/annurev-ento-120811-153542 23317046

[B7] Hall D. G. Richardson M. L. Ammar E. D. Halbert S. E. (2013). Asian citrus psyllid, *Diaphorina citri*, vector of citrus huanglongbing disease. *Entomol. Exp. Appl.* 146 207–223. 10.1111/eea.12025

[B8] Hogenhout S. A. Ammar E. D. Whitfield A. E. Redinbaugh M. G. (2008). Insect vector interactions with persistently transmitted viruses. *Annu. Rev. Phytopathol.* 46 327–359. 10.1146/annurev.phyto.022508.092135 18680428

[B9] Hogenhout S. A. Bos J. I. (2011). Effector proteins that modulate plant-insect interactions. *Curr. Opin. Plant Biol.* 14 422–428. 10.1016/j.pbi.2011.05.003 21684190

[B10] Huang H. J. Liu C. W. Huang X. H. Zhou X. Zhuo J. C. Zhang C. X. (2016). Screening and functional analyses of *Nilaparvata lugens* salivary proteome. *J. Proteome Res.* 15 1883–1896. 10.1021/acs.jproteome.6b00086 27142481

[B11] Huang H. J. Lu J. B. Li Q. Bao Y. Y. Zhang C. X. (2018). Combined transcriptomic/proteomic analysis of salivary gland and secreted saliva in three planthopper species. *J. Proteomics* 172 25–35. 10.1016/j.jprot.2017.11.003 29109049

[B12] Ji R. Ye W. Chen H. Zeng J. Li H. Yu H. (2017). A salivary endo-beta-1,4-glucanase acts as an effector that enables the brown planthopper to feed on rice. *Plant. Physiol.* 173 1920–1932. 10.1104/pp.16.01493 28126846 PMC5338667

[B13] Ji R. Yu H. X. Fu Q. Chen H. D. Ye W. F. Li S. H. (2013). Comparative transcriptome analysis of salivary glands of two populations of rice brown planthopper, *Nilaparvata lugens*, that differ in virulence. *PLoS One* 8:e79612. 10.1371/journal.pone.0079612 24244529 PMC3828371

[B14] Killiny N. Hijaz F. Ebert T. A. Rogers M. E. (2017). A plant bacterial pathogen manipulates its insect vector’s energy metabolism. *Appl. Environ. Microbiol.* 83 e3005–e3016. 10.1128/AEM.03005-16 28039132 PMC5311395

[B15] Li X. C. Schuler M. A. Berenbaum M. R. (2007). Molecular mechanisms of metabolic resistance to synthetic and natural xenobiotics. *Annu. Rev. Entomol.* 52 231–253. 10.1146/annurev.ento.51.110104.151104 16925478

[B16] Liu X. Q. Jiang H. B. Xiong Y. Peng P. Li H. F. Yuan G. R. (2019). Genome-wide identification of ATP-binding cassette transporters and expression profiles in the Asian citrus psyllid, *Diaphorina citri*, exposed to imidacloprid. *Comp. Biochem. Physiol. D Genomics Proteomics* 30 305–311. 10.1016/j.cbd.2019.04.003 31004937

[B17] Liu X. Q. Zhou H. Y. Zhao J. Hua H. X. He Y. P. (2016). Identification of the secreted watery saliva proteins of the rice brown planthopper, *Nilaparvata lugens* (Stal) by transcriptome and Shotgun LC-MS/MS approach. *J. Insect Physiol.* 89 60–69. 10.1016/j.jinsphys.2016.04.002 27080912

[B18] Livak K. J. Schmittgen T. D. (2001). Analysis of relative gene expression data using real-time quantitative PCR and the 2^−ΔΔ^^CT^ method. *Methods* 25 402–408. 10.1006/meth.2001.1262 11846609

[B19] Love M. I. Huber W. Anders S. (2014). Moderated estimation of fold change and dispersion for RNA-seq data with DESeq2. *Genome Biol.* 15:550. 10.1186/s13059-014-0550-8 25516281 PMC4302049

[B20] Ma J. Chen T. F. Wu S. Y. Yang C. Z. Bai M. X. Shu K. (2019). iProX: an integrated proteome resource. *Nucleic Acids Res.* 47 D1211–D1217. 10.1093/nar/gky869 30252093 PMC6323926

[B21] Matsumoto Y. Suetsugu Y. Nakamura M. Hattori M. (2014). Transcriptome analysis of the salivary glands of *Nephotettix cincticeps* (Uhler). *J. Insect Physiol.* 71 170–176. 10.1016/j.jinsphys.2014.10.010 25450428

[B22] Mutti N. S. Louis J. Pappan L. K. Pappan K. Begum K. Chen M. S. (2008). A protein from the salivary glands of the pea aphid, *Acyrthosiphon pisum*, is essential in feeding on a host plant. *Proc. Natl. Acad. Sci. U.S.A.* 105 9965–9969. 10.1073/pnas.0708958105 18621720 PMC2481341

[B23] Naessens E. Dubreuil G. Giordanengo P. Baron O. L. Minet-Kebdani N. Keller H. (2015). A secreted MIF cytokine enables aphid feeding and represses plant immune responses. *Curr. Biol.* 25 1898–1903. 10.1016/j.cub.2015.05.047 26119751

[B24] Nicholson S. J. Hartson S. D. Puterka G. J. (2012). Proteomic analysis of secreted saliva from Russian wheat aphid (*Diuraphis noxia* Kurd.) *biotypes that differ in virulence to wheat*. *J. Proteomics* 75 2252–2268. 10.1016/j.jprot.2012.01.031 22348819

[B25] Picelli S. Bjorklund A. K. Faridani O. R. Sagasser S. Winberg G. Sandberg R. (2013). Smart-seq2 for sensitive full-length transcriptome profiling in single cells. *Nat. Methods* 10 1096–1098. 10.1038/nmeth.2639 24056875

[B26] Saikhedkar N. Summanwar A. Joshi R. Giri A. (2015). Cathepsins of lepidopteran insects: Aspects and prospects. *Insect Biochem. Mol. Biol.* 64 51–59. 10.1016/j.ibmb.2015.07.005 26210259

[B27] Serteyn L. Francis F. (2019). Insight into salivary gland proteomes of two polyphagous stink bugs: *Nezara viridula* L. *and Halyomorpha halys Stål*. *Proteomics* 19:1800436. 10.1002/pmic.201800436 30793498

[B28] Sharma A. Khan A. N. Subrahmanyam S. Raman A. Taylor G. S. Fletcher M. J. (2014). Salivary proteins of plant-feeding hemipteroid - implication in phytophagy. *Bull. Entomol. Res.* 104 117–136. 10.1017/S0007485313000618 24280006

[B29] Su Y. L. Li J. M. Li M. Luan J. B. Ye X. D. Wang X. W. (2012). Transcriptomic analysis of the salivary glands of an invasive whitefly. *PLoS One* 7:e39303. 10.1371/journal.pone.0039303 22745728 PMC3379992

[B30] Tian F. J. Wang Z. B. Li C. F. Liu J. L. Zeng X. N. (2019). UDP-Glycosyltransferases are involved in imidacloprid resistance in the Asian citrus psyllid, *Diaphorina citri* (Hemiptera: Lividae). *Pestic. Biochem. Physiol.* 154 23–31. 10.1016/j.pestbp.2018.12.010 30765053

[B31] Tiwari S. Pelz-Stelinski K. Mann R. S. Stelinski L. L. (2011). Glutathione transferase and cytochrome P450 (general oxidase) activity levels in C*andidatus* Liberibacter Asiaticus-infected and uninfected Asian citrus psyllid (Hemiptera: Psyllidae). *Ann. Entomol. Soc. Am.* 104 297–305. 10.1603/an10128

[B32] Trapnell C. Pachter L. Salzberg S. L. (2009). TopHat: discovering splice junctions with RNA-Seq. *Bioinformatics* 25 1105–1111. 10.1093/bioinformatics/btp120 19289445 PMC2672628

[B33] van Bel A. J. Will T. (2016). Functional evaluation of proteins in watery and gel saliva of aphids. *Front. Plant Sci.* 7:1840. 10.3389/fpls.2016.01840 28018380 PMC5156713

[B34] Weintraub P. G. Beanland L. (2006). Insect vectors of phytoplasmas. *Annu. Rev. Entomol.* 51 91–111. 10.1146/annurev.ento.51.110104.151039 16332205

[B35] Xu H. X. Qian L. X. Wang X. W. Shao R. X. Hong Y. Liu S. S. (2019). A salivary effector enables whitefly to feed on host plants by eliciting salicylic acid-signaling pathway. *Proc. Natl. Acad. Sci. U.S.A.* 116 490–495. 10.1073/pnas.1714990116 30584091 PMC6329982

[B36] Yang Z. X. Ma L. Francis F. Yang Y. Chen H. Wu H. X. (2018). Proteins identified from saliva and salivary glands of the Chinese gall aphid *Schlechtendalia chinensis*. *Proteomics* 18:1700378. 10.1002/pmic.201700378 29577599

[B37] Yu H. Z. Huang Y. L. Li N. Y. Xie Y. X. Zhou C. H. Lu Z. J. (2019). Potential roles of two cathepsin genes, DcCath-L and DcCath-O in the innate immune response of *Diaphorina citri*. *J. Asia-Pac. Entomol.* 22 1060–1069. 10.1016/j.aspen.2019.05.010

[B38] Yu X. D. Killiny N. (2018). The secreted salivary proteome of Asian citrus psyllid *Diaphorina citri*. *Physiol. Entomol.* 43 324–333. 10.1111/phen.12263

