# Supplementary figures and images for: A Transcriptomic and Proteomic Analysis of the Diaphorina citri Salivary Glands Reveals Genes Responding to Candidatus Liberibacter asiaticus
Source: Front Physiol. 2020 Sep 25;11:582505. doi: 10.3389/fphys.2020.582505 (PMC7546269; doi:10.3389/fphys.2020.582505)

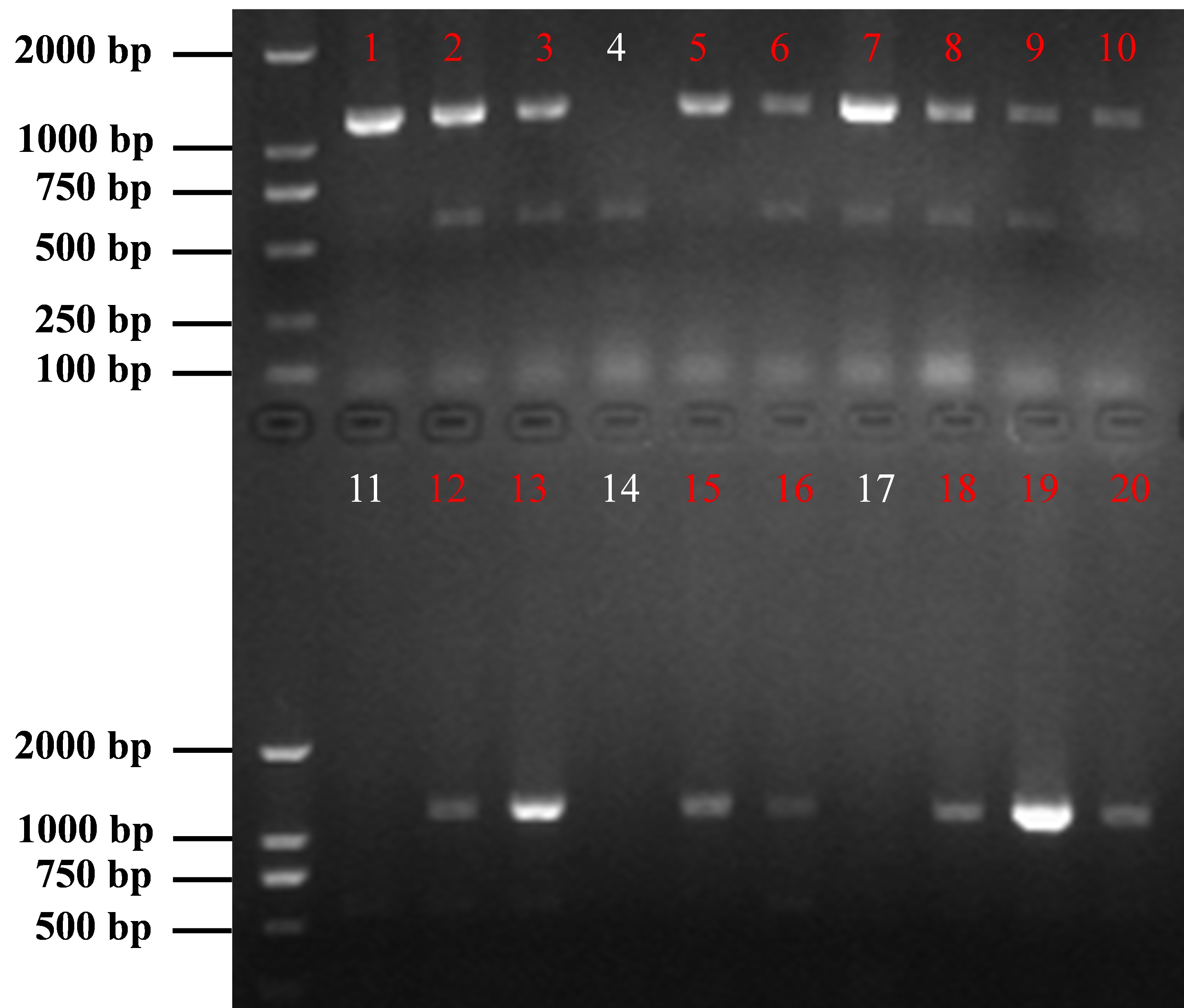

Supplement: FIGURE S1 — RT-PCR detection the proportion of Candidatus Liberibacter asiaticus (CLas) in CLas-infected Diaphorina citri population. The thorax from a single D. citri was dissected, then genomic DNA was isolated, and RT-PCR was performed using the OI1/OI2c primer (forward primer 5′-GCGCGTATGCAATACGAGCGGCA-3′ and reverse primer 5′-GCCTCGCGACTTCGCAACCCAT-3′). Twenty D. citri were randomly selected to check HLB, and 16 ones exhibited a clear OI1 band with 1160 bp in length. [file Image_1.JPEG]
