# Supplementary material for: A Transcriptomic and Proteomic Analysis of the Diaphorina citri Salivary Glands Reveals Genes Responding to Candidatus Liberibacter asiaticus
Source: Front Physiol. 2020 Sep 25;11:582505. doi: 10.3389/fphys.2020.582505 (PMC7546269; doi:10.3389/fphys.2020.582505)
Supplement: TABLE S1 — Primers used in RT-qPCR for validation of DEGs. [file Data_Sheet_1.docx]

Table S1. Primers used in RT-qPCR for validation of differentially expressed genes (DEGs).

| Primer names | Forward primer (5'−3') | Reverse primer (5'−3') | Efficiency (%) |
| --- | --- | --- | --- |
| *DcitCathepsin-F5*  *DcitUGT*  *DcitGST*  *DcitSpondin-1*  *DcitPOD*  *Dcit**Aminopeptidase*  *DcitCYP6KB1*  *DcitCathepsin-F*  *DcitCRISP*  *DcitCathepsin-B*  *Actin1*  *GAPDH2* | AAAGTGAACGCCTTGGTGTC CACCGCCCAATATTTCAGCA  TGATGCCCTGTATCCCAAGG GGATAAGCAACACGAGGCAG  CATTCCCATACCCGCCAATG TTGAAAGACGGCGAAACTGG  CACCAACCTTTACCTCGGGA CGCCGATTATTTCCGTGGTT  CAGTTCTGTGCTGCATTCCT  GGACGCCTACATCGATCAGA  TGTGACGAAGAAGTTGCTGC  CATGGCAAGTTCAACGGTGA | TTCATCCGTCTCCTTGCCAT GCCTGTTCTTTGTCCAACGT  GTCGCCCGTAATGAACTTCC ACATCCCTTTCATCGGCTCA  GTTGGAGCGCAGGGATTTAG  GAAATTGGTGGAGTGGCTGG ACCAAACACTGTAGAGCCGA  ACACATCTTGCAACAGCTCG  AGTTAGGACAACAGCGCTTG  CAGTTAGGCCATTGTTCCCG  TGGGGTATTTCAGGGTCAGG  CGATGCCTTCTCAATGGTGGA | 91.1  93.7  105  110.1  105  105  110.9  104.9  109.3  110  93.9  102.5 |
