# Supplementary material for: A Transcriptomic and Proteomic Analysis of the Diaphorina citri Salivary Glands Reveals Genes Responding to Candidatus Liberibacter asiaticus
Source: Front Physiol. 2020 Sep 25;11:582505. doi: 10.3389/fphys.2020.582505 (PMC7546269; doi:10.3389/fphys.2020.582505)
Supplement: TABLE S2 — Differentially expressed genes upon to CLas infection involved in the ribosome, the insecticide resistance, the immune response and the digestion in comparison with CLas-infected SG and CLas-free SG. [file Data_Sheet_2.docx]

Table S2 Differentially expressed genes upon to CLas infection involved in the ribosome, the insecticide resistance, the immune response and the digestion in comparison with CLas-infected SG and CLas-free SG.

| Gene ID | Log_2_Fold | *P* adj | Gene_description |
| --- | --- | --- | --- |
| **Ribosome** |  |  |  |
| DcitrP019760.1 | 11.41 | 6.24E-06 | 60S ribosomal protein L18a |
| DcitrP081710.1 | -5.77 | 1.50E-06 | 39S ribosomal protein L51 |
| DcitrP088465.1 | -1.31 | 0.012 | 39S ribosomal protein L30 |
| DcitrP093430.1 | 3.71 | 2.20E-11 | Ribosomal protein L27A |
| DcitrP080835.1 | 4.21 | 0.014 | 39S ribosomal protein L53 |
| DcitrP091755.1 | 1.45 | 8.48E-03 | 40S ribosomal protein SA |
| **Insecticide resistance** |  |  |  |
| DcitrP019570.1  DcitrP020605.1  DcitrP093380.1  DcitrP097555.1  DcitrP069555.1  DcitrP045800.1  DcitrP045795.1  DcitrP045790.1 | 1.77  1.55  -1.89  -1.36  -1.40  -2.07  -2.15  -3.08 | 4.97E-05  4.27E-04  4.11E-04  0.029  3.86E-03  0.034  7.52E-03  4.77E-03 | Glutathione *S*-transferase  Glutathione *S*-transferase  Glutathione *S*-transferase  CYP3172A  CYP307B  CYP301A1  CYP4C67  CYP6KB1 |
| DcitrP084155.1 | 7.66 | 2.78E-04 | UDP-glucuronosyltransferase |
| DcitrP038140.1 | 2.05 | 2.14E-03 | UDP-glucuronosyltransferase |
| DcitrP069135.1 | -2.40 | 0.013 | UDP-glucuronosyltransferase |
| **Immune response** |  |  |  |
| DcitrP082300.1 | 7.95 | 7.52E-03 | Unknown protein |
| DcitrP103380.1 | -1.13 | 0.043 | Aminopeptidase |
| DcitrP011585.1 | -2.52 | 0.022 | Aminopeptidase |
| DcitrP009135.1 | 1.85 | 9.60E-05 | Peroxidase like |
| DcitrP053520.1 | 1.61 | 5.36E-04 | Peroxidase like |
| DcitrP016435.1 | 1.16 | 0.04 | Lysozyme i-type |
| DcitrP050505.1 | 1.38 | 0.043 | Spondin-1 |
| DcitrP033280.1  DcitrP009550.1  DcitrP029635.1 | 9.99  -11.93  -9.90 | 8.73E-03  2.48E-06  1.94E-06 | Beat-IIa  Cathepsin B-like protein 8  Cathepsin B-like cysteine proteinase 5 |
| **Digestion** |  |  |  |
| DcitrP009555.1 | -5.69 | 0.033 | Cathepsin F Like cysteine proteinase |
| DcitrP010820.1 | -10.55 | 5.26E-05 | Cathepsin F-like protein 5 |
| DcitrP016100.1 | -10.18 | 9.65E-05 | Cathepsin F-like protein 5 |
| DcitrP068425.1 | -8.75 | 1.16E-06 | Cathepsin F-like protein 5 |
| DcitrP077305 | 9.17 | 0.03 | Cathepsin F-like protein 5 |
| DcitrP068390.1 | -4.42 | 0.015 | Cathepsin W-like protein 2 |
| DcitrP072815.1 | -13.27 | 1.13E-07 | Maltase |
| DcitrP091865.1 | -6.93 | 5.22E-03 | Maltase |
| DcitrP036010.1 | -8.12 | 2.35E-03 | Salivary cysteine-rich peptide |
| DcitrP055350.1 | -10.37 | 3.47E-10 | Salivary secreted protein |
| DcitrP072075.1 | -1.68 | 0.019 | Mucin-17-like |
