# Supplementary material for: A Transcriptomic and Proteomic Analysis of the Diaphorina citri Salivary Glands Reveals Genes Responding to Candidatus Liberibacter asiaticus
Source: Front Physiol. 2020 Sep 25;11:582505. doi: 10.3389/fphys.2020.582505 (PMC7546269; doi:10.3389/fphys.2020.582505)
Supplement: TABLE S7 — The information of selected 10 DEGs used in RT-qPCR validation. [file Data_Sheet_7.docx]

Table S6 Information on the 10 selected DEGs used in RT-qPCR validation.

| Gen ID | Log2Fold | Gene_description |
| --- | --- | --- |
| DcitrP077305.1 | 9.17 | Cathepsin F-like protein 5 |
| DcitrP084155.1 | 7.66 | UDP-glucuronosyltransferase |
| DcitrP020605.1 | 1.55 | Glutathione S-transferase |
| DcitrP053520.1 | 1.61 | Peroxidase like |
| DcitrP050505.1 | 1.38 | Spondin-1 |
| DcitrP011585.1 | -2.52 | Aminopeptidase |
| DcitrP045790.1 | -3.08 | CYP6KB1-RA |
| DcitrP009555.1 | -5.69 | Cathepsin F Like cysteine proteinase partial |
| DcitrP036010.1 | -8.12 | Cysteine-rich salivary peptide |
| DcitrP009550.1 | -11.93 | Cathepsin B-like protein 8 |
